# Predictive factors for dissection-free sentinel node micrometastases in early oral squamous cell carcinoma

**DOI:** 10.1038/s41598-023-33218-8

**Published:** 2023-04-15

**Authors:** Takashi Matsuzuka, Kiyoaki Tsukahara, Seiichi Yoshimoto, Kazuaki Chikamatsu, Akihiro Shiotani, Isao Oze, Yoshiko Murakami, Takeshi Shinozaki, Yuichiro Enoki, Shinichi Ohba, Daisuke Kawakita, Nobuhiro Hanai, Yusuke Koide, Michi Sawabe, Yusuke Nakata, Yujiro Fukuda, Daisuke Nishikawa, Gaku Takano, Takahiro Kimura, Keisuke Oguri, Hitoshi Hirakawa, Yasuhisa Hasegawa

**Affiliations:** 1 Department of Head and Neck Surgery - Otorhinolaryngology, Asahi University Hospital, 3-23 Hashimotocou, Gifu, 500-8523 Japan; 2 Department of Otorhinolaryngology Head and Neck Surgery, Tokyo Medical University, Tokyo, Japan; 3 Department of Head and Neck Surgery, National Cancer Center Hospital, Tokyo, Japan; 4 Department of Otolaryngology Head and Neck Surgery, Gunma University School of Medicine, Maebashi, Japan; 5 Department of Otolaryngology Head and Neck Surgery, National Defense Medical College, Tokorozawa, Japan; 6 Division of Cancer Epidemiology and Prevention, Aichi Cancer Center Research Institute, Nagoya, Japan; 7 Department of Diagnostic Pathology, Nagoya Medical Center, Nagoya, Japan; 8 Department of Head and Neck Surgery, National Cancer Center Hospital East, Kashiwa, Japan; 9 Department of Head and Neck Oncology / Ear, Nose and Throat, Saitama Medical University International Medical Center, Saitama, Japan; 10 Department of Otorhinolaryngology, Juntendo University Graduate School of Medicine, Tokyo, Japan; 11 Department of Otorhinolaryngology, Head and Neck Surgery, Nagoya City University Graduate School of Medical Sciences, Nagoya, Japan; 12 Department of Head and Neck Surgery, Aichi Cancer Center Hospital, Nagoya, Japan; 13 Department of Otolaryngology Head and Neck Surgery, Japan Community Health Care Organization Chukyo Hospital, Nagoya, Japan; 14 Department of Otorhinolaryngology, Shiga University of Medical Science, Otsu, Japan; 15 Department of Otolaryngology Head and Neck Surgery, Kawasaki Medical School, Kurashiki, Japan; 16 Department of Otorhinolaryngology, Kindai University Nara Hospital, Nara, Japan; 17 Department of Otorhinolaryngology, Nagoya City University West Medical Center, Nagoya, Japan; 18 Department of Otolaryngology - Head and Neck Surgery, Nara Medical University, Kashihara, Japan; 19 Department of Otorhinolaryngology, Konan Kosei Hospital, Konan, Japan; 20 Department of Otorhinolaryngology, Head and Neck Surgery, University of the Ryukyus Faculty of Medicine, Okinawa, Japan

**Keywords:** Cancer, Oral diseases, Oncology

## Abstract

This sentinel node (SN) biopsy trial aimed to assess its effectiveness in identifying predictive factors of micrometastases and to determine whether elective neck dissection is necessary in oral squamous cell carcinoma. This retrospective study included 55 patients from three previous trials, with positive SNs. The relationship between the sizes of the metastatic focus and metastasis in non-sentinel node (NSN) was investigated. Four of the 55 largest metastatic focus were isolated tumor cells, and the remaining 51 were ranged from 0.2 to 15 mm, with a median of 2.6 mm. The difference of prevalence between 46 negative- and 9 positive-NSN was statistically significant with regard to age, long diameter of primary site and number of cases with regional recurrence. In comparing the size of largest metastatic focus dividing the number of positive SN, with metastaic focus range of < 3.0 mm in one-positive SN group, there were 18 (33%) negative-NSN and no positive-NSN. Regarding prognosis, 3-year overall survival rate of this group (n = 18) and other (n = 37) were 94% and 73% (*p* = 0.04), and 3-year recurrence free survival rate of this group and other were 94% and 51% (*p* = 0.03), respectively. Absolutely a further prospective clinical trial would be needed, micrometastases may be defined as solitary SN metastasis with < 3.0 mm of metastatic focus, and approximately 33% of neck dissections could be avoided using these criteria.

## Introduction

Elective neck dissection (END) is recommended as a treatment strategy for early oral squamous cell carcinoma (OSCC). It aims to prevent metastasis to the cervical lymph nodes, thus improving the chance of survival. Although END has low morbidity and mortality, the former is inevitable owing to the nature of the procedure; moreover, a large number of such unnecessary procedures are performed^1^. Sentinel node (SN) biopsy has been explored in clinical trials as a tool for the management of early OSCC, which may help avoid unnecessary ENDs^2^. In Japan, a national multi-facilities group was organized in 2009 to acquire insights on SN navigation surgery and its viability for OSCC. Briefly, radioisotope scanning, by itself or in combination with indocyanine, was used to detect SNs. These were examined with multi-slice frozen section analysis intraoperatively; patients with positive SNs underwent one-stage or backup neck dissection procedures.

In the clinical pathology of breast cancer, the volume of metastasis in the lymph node can be fit into one of three categories: isolated tumor cells (ITC), micrometastasis, and macrometastasis. Treatment guidelines depend on how each case is classified. In contrast, there is no pathological classification for head and neck cancer, and clinical treatment is based solely on the presence or absence of lymph node metastases. SN biopsy allows assessment of lymph nodes beyond this level, which may, in turn, allow for early detection of micrometastases.

Because the properties of the cancerous cells from specific tissues are different, the diagnostic criteria used for breast cancer cannot be applied to oral cancer. We hypothesize that if the status of SN metastases is known early enough, non-sentinel node (NSN) metastases can be avoided. Thus, the present study aimed to investigate the relationship between the status of SN and NSN to assess the effectiveness of SN biopsy for patients with OSCC. The biopsy should be adequate for identifying micrometastasis, thus helping determine non-invasive treatment for micrometastasis when END is unnecessary.

## Methods

This retrospective study builds on data from previous phase II (A, B) and phase III (C) trials^3–5^. Details of the SN detection by radioisotope scanning and SN biopsy are described in the respective reports. A total of 162 cases of SN biopsy were registered (trial A: 42 cases, B: 18 and C: 104). After the biopsy, all 60 of the phase II cases underwent backup neck dissection, and 102 of the phase III cases underwent SN navigation surgery. Of the 162 cases, 62 had pathologically positive SN biopsy results. The size of the primary region and the size of metastatic focus in the positive SNs were additionally registered after reexamination by the Ethical Review Board of the Cancer Center (Feb. 14, 2017/ No. 2017-293). This research has been performed following the relevant guidelines and regulations, including the Declaration of Helsinki. We were unable to re-examine or obtain consent from seven patients. There were four patients in facilities which did not participate in this supplementary study, and three patients whose measurement of metastatic focus were inability. Finally, 55 patients (study A; 15 cases, B; 6 and C; 34, mean age: 58.5 ± 16.3 years old) were registered were considered as participants in this study.

Size of metastatic focus was measured microscopically as the maximum longitudinal diameters of positive lesions, based on AE1/3 cytokeratin staining. In cases with more than one-positive SNs, size of largest metastatic focus was taken from the biggest positive lesion. Cases in which metastatic focus were single cells or clusters of cells of < 0.2 mm in diameter, were registered non-numerically as ITC. The metastatic status of NSN in each case was classified as negative or positive NSN, and compared between both groups while considering the number of positive SN. Positive-NSN was defined, during surgery, as the presence of cancer cells within NSN at the level of therapeutic neck dissection. Cases except positive-NSN were considered to have negative-NSN.

The relationship between size of largest metastatic focus and NSN metastasis was investigated. For continuous data, we used the Student’s t-test to compare the two groups. We used the Pearson's chi-square test and Fisher’s exact test for comparisons of discrete variables. Additionally, the clinical findings and tumor characteristics predicted to have an impact on NSN metastasis were analyzed using logistic analysis. StatView® version 5.0 (SAS Institute Inc., USA) and Microsoft Excel^®^ 2019 (Microsoft Corporation, USA) were used for statistical analyses. A p-value less than 0.05 was considered statistically significant.

## Results

Patient’s characteristics are listed in Table 1. Four of the 55 largest metastatic focus were ITC, while the size of remaining 51 largest metastatic focus ranged from 0.2 to 15 mm; the median size of largest metastatic focus was 2.6 mm. Of these 55 patients, 9 (16%, mean age: 68.8 ± 7.69 years old) had pathological metastasis in NSNs. Thus, the number of negative-NSN and positive-NSN were considered to be 46 (84%) and 9 (16%), respectively. The difference of prevalence between negative- and positive-NSN was statistically significant with regard to age, long diameter of primary site and number of cases with regional recurrence (Table 1). Extranodal extension was observed in four cases of positive SN, two cases showed negative-NSN whereas the others showed positive-NSN, and size of largest metastatic focus in each case was 4.0, 4.7, 3.0 and 12 mm, respectively. All four had underwent adjuvant radiotherapy.

**Table 1:** Characteristics of 55 cases with sentinel node metastasis and comparison with and without non-sentinel node metastasis.

	Total (n = 55)	Negative-NSN (n = 46)	Positive-NSN (n = 9)	*p*
Age	58.5 ± 16.3	56.5 ± 16.8	68.8 ± 7.7	0.04
Male/female	40/15	34/12	6/3	NS
Tongue/oral floor/Gingiva	48/6/1	40/6/0	8/0/1	NS
8th pT1/pT2/pT3/pT4	10/22/20/3	9/19/17/1	1/3/3/2	NS
Depth of invasion of primary site	8.91 ± 4.29	8.86 ± 4.15	9.21 ± 5.23	NS
Long diameter of primary site	24.1 ± 9.81	22.3 ± 8.34	33.6 ± 11.7	0.03
Number of SN	3.51 ± 1.47	3.56 ± 1.34	3.22 ± 2.11	NS
Number of positive SN	1.69 ± 0.81	1.63 ± 0.83	2.00 ± 0.71	NS
Size of largest metastatic focus	3.75 ± 3.87	3.72 ± 4.02	3.91 ± 3.16	NS
Number of cases underwent adjuvant radiation	4	3	1	NS
3-year overall survival rate	79.6%	77.8%	88.9%	NS
3-year recurrence free survival rate	64.9%	69.1%	44.4%	NS
3-year regional recurrence free survival rate	81.0%	84.0%	66.7%	NS
Number of cases with regional recurrence	10	6	4	0.05

*NSN* Non-sentinel node, *8th* TNM classification according to 8th edition of the Union for International Cancer Control, *SN* Sentinel node, *NS* Not significant.

Comparing the size of largest metastatic focus between negative- and positive-NSN, the median size of largest metastatic focus in the 46 negative-NSN and the 9 positive-NSN was 2.4 mm (ITC: 4 cases, numeric range: 0.2–15 mm) and 3.5 mm (ITC: 1 case, numeric range: 0.9–10 mm), respectively.

**Figure 1:**
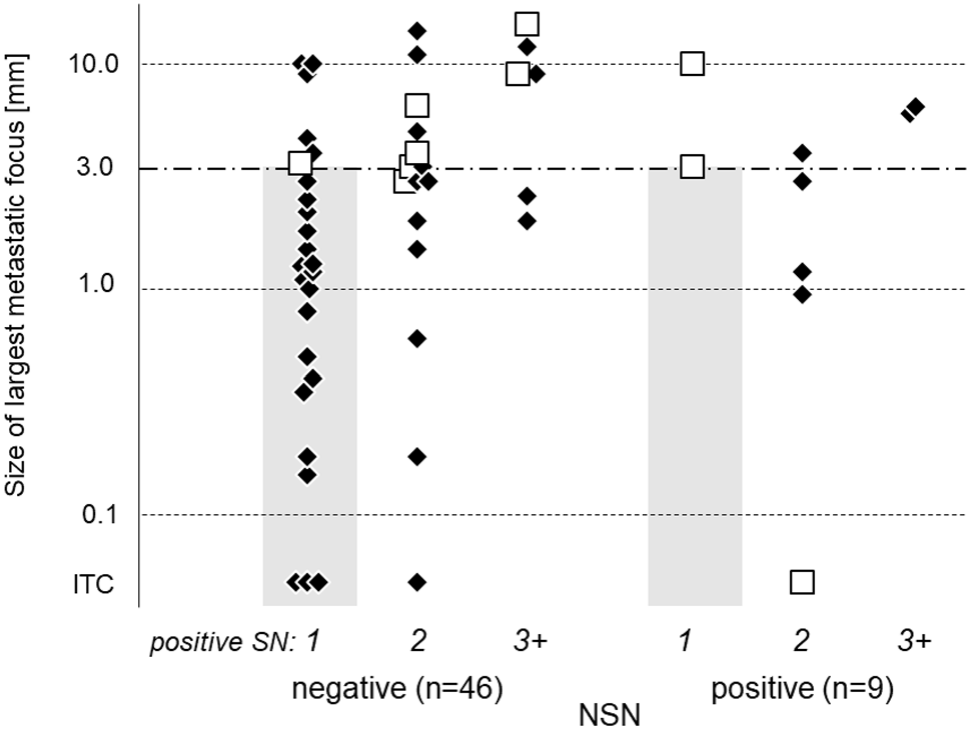
Comparison of size of largest metastatic focus between negative-NSN and positive-NSN dividing the number of positive SN. *White square* in the figure shows regional recurrence. With a size of largest metastatic focus range of 3.0 mm or less in one-positive SN group (gray area), there were 18 (33%) negative-NSN and no positive-NSN. 3-year overall survival rate of this group and other were 94.1% and 73.0% (*p* = 0.04), and 3-year recurrence free survival rate of this group and other were 94.1% and 51.2% (*p* = 0.03), respectively. *NSN* Non-sentinel node, *SN* Sentinel node, *ITC* Isolated tumor cells, *white square* Regional recurrence.

If positive SN cases are grouped according to the number of affected nodes, that is, one, two, and three and more, the rates of positive-NSN were 7% (2/27), 25% (5/20) and 25% (2/8), respectively. Figure 1 shows a comparison o size of largest metastatic focus between negative and positive-NSN with respect to the number of positive SN. In one-positive group, there were 25 negative-NSN and two positive-NSN, the median size of largest metastatic focus of negative-NSN was 1.2 mm (ITC: 3 cases, numeric range: 0.2 to 10 mm) and the size of positive NSN were 3.5 and 10 mm. With a size of largest metastatic focus range of 3.0 mm or less in one-positive SN group, there were 18 (33%) negative-NSN and no positive-NSN. Regarding prognosis of this group, one patient died with primary recurrence and the others alive without relapse during the follow-up period. 3-year overall survival rate of this group and other were 94.1% and 73.0% (*p* = 0.04), and 3-year recurrence free survival rate of this group and other were 94.1% and 51.2% (*p* = 0.03), respectively.

## Discussion

Lymph node metastasis is an important factor in the prognosis of OSCC. In the management of the neck for early OSCC, despite the morbidity associated with END, it is strongly suggested that it be routinely performed to increase both the local control of metastasis and the chances of survival^6–8^. The purpose of SN biopsy in OSCC is to distinguish cases without metastasis in the SN, in which END is avoidable^9^. SN biopsy is also associated with better postoperative mobility and shoulder function when compared to END^10^. According to the European multicentre study, the presence of positive NSN was a factor for poor prognosis^11^. In western countries, it is recommended that OSCC patients be offered cervical node testing by neither END or SN biopsy. Japan’s Pharmaceutical Affairs Law does not allow the use of tracers, such as indocyanine, tilmanocept, and phytate, to detect SN in OSCC patient. We have promoted their acceptance for clinical use in early OSCC and laryngo-pharyngeal squamous cell carcinoma based on domestic multi-institutional joint research^12^. Trial A was a phase II trial with 57 OSCC cases, performed to examine the feasibility of SN biopsy with back up END^3^. Trial B was a phase II trial with 20 OSCC cases, aimed to examine the diagnostic accuracy of indocyanine green (ICG) fluorescence navigated SNBs in comparison to the radioisotope method^4^. Trial C, a phase III randomized trial including 271 OSCC cases, aimed to compare SN navigation surgery with END, found that the 3-year overall and disease-free survival rates did not differ between the SN navigation surgery (87.9%, 78.7%) and END groups (86.6%, 75.0%). The scores of neck functionality in the SN navigation surgery group were significantly better than those in the END group. SN navigation surgery may replace END without a survival disadvantage and reduce postoperative neck disability in patients with early-stage OSCC^5^.

In the treatment of early breast cancer, SN biopsy has been used routinely for several decades; management strategies based on its results have changed. Formerly, in cases with positive SN, axillary node dissection (AD) used to be a common treatment to reduce the risk of recurrence. Later studies concluded that AD was not recommended routinely because the disease-free survival of patients with positive SNs treated with SN biopsy alone was almost identical to that of patients with negative SN^13^. SN dissection alone and AD also offered similar survival rates^14^, whereas lymph node radiation and AD offered similar recurrence and survival rates, with a higher incidence of lymphedema after AD^15^. While pathological classification of SN is mentioned as an independent predictor of the involvement of NSN, this depends significantly and exclusively on macro-metastatic SN^16^. Some studies suggest that micrometastases and ITC found in the SN are a sign of another, presumably systemic disease, which may not justify an aggressive treatment approach^17–21^. Micrometastases is considered an important factor for predicting the prognosis and selecting a surgical strategy^22,23^.

Although the pathological status was defined for breast cancer, it has been reported that the SN status has a significant impact on survival, depending on whether it is negative, ITC, micrometastases or macrometastasis^24^. The spread of SN biopsy for OSCC was expected to result in an increase in the reports of micrometastases as an artifact of increased testing. Therefore, it is important to prepare for a concept of the micrometastases in the treatment of OSCC. It is assumed that micrometastases for OSCC is the condition when cancer cell infiltration in the SN is too small to extend to the other regional nodes; in these cases, SN lymphadectomy may be considered as an adequate treatment. To define the tissue characteristics of micrometastases in OSCC could help in the development of minimally invasive treatments^25^. In the present study, if metastatic focus was small enough and positive SN was not accompanied by positive NSN, hidden NSN metastases tended not to occur. Multiple positive SN was reported as one of the predictive factors for NSN metastasis^26^. Generally, metastasis in multiple lymph nodes is a factor for poor prognosis. The plural positive SN should also be excluded from a definition of the micrometastases. Our suggestion is for micrometastases to be defined as the condition where SN metastasis is solitary and metastatic focus is less than 3.0 mm, with a margin of error yet to be defined. Using these criteria, approximately 33% of neck dissections in OSCC patients with positive SN could be avoided. To verify this hypothesis, a further prospective clinical trial would be needed. In melanoma primary tumor burden and the distribution in the positive SN whether metastasis foci were subcapsular or parenchymal are also predictive of non-SLN metastases^27^. In planning and executing further trial, various related to lymph node metastasis, such as the number of lymph nodes examined, should be included.

## Data Availability

Since patient data cannot be made available, no access details can be provided. Any other requests for information should be made to the corresponding author.
